# Exercise-like effects by Estrogen-related receptor-gamma in muscle do not prevent insulin resistance in db/db mice

**DOI:** 10.1038/srep26442

**Published:** 2016-05-25

**Authors:** Pierre-Marie Badin, Isabelle K. Vila, Danesh H. Sopariwala, Vikas Yadav, Sabina Lorca, Katie Louche, Eun Ran Kim, Qingchun Tong, Min Sup Song, Cedric Moro, Vihang A. Narkar

**Affiliations:** 1 Metabolic and Degenerative Diseases, Institute of Molecular Medicine, The University of Texas McGovern Medical School, Houston, USA; 2 Molecular and Cellular Oncology, The University of Texas MD Anderson Cancer Center, Houston, USA; 3 Institut National de la Santé et de la Recherche Médicale, Inserm UMR 1048, Institute of Metabolic and Cardiovascular Diseases, and Paul Sabatier University, Toulouse, France; 4 Graduate School of Biomedical Sciences at The University of Texas Health Science Center at Houston, Houston, USA; 5 Integrative Biology and Pharmacology, The University of Texas McGovern Medical School, Houston, USA.

## Abstract

Dissecting exercise-mimicking pathways that can replicate the benefits of exercise in obesity and diabetes may lead to promising treatments for metabolic disorders. Muscle estrogen-related receptor gamma (ERRγ) is induced by exercise, and when over-expressed in the skeletal muscle mimics exercise by stimulating glycolytic-to-oxidative myofiber switch, mitochondrial biogenesis and angiogenesis in lean mice. The objective of this study was to test whether muscle ERRγ in obese mice mitigates weight gain and insulin resistance. To do so, ERRγ was selectively over-expressed in the skeletal muscle of obese and diabetic db/db mice. Muscle ERRγ over-expression successfully triggered glycolytic-to-oxidative myofiber switch, increased functional mitochondrial content and boosted vascular supply in the db/db mice. Despite aerobic remodeling, ERRγ surprisingly failed to improve whole-body energy expenditure, block muscle accumulation of triglycerides, toxic diacylglycerols (DAG) and ceramides or suppress muscle PKCε sarcolemmal translocation in db/db mice. Consequently, muscle ERRγ did not mitigate impaired muscle insulin signaling or insulin resistance in these mice. In conclusion, obesity and diabetes in db/db mice are not amenable to selective ERRγ-directed programming of classic exercise-like effects in the skeletal muscle. Other biochemical pathways or integrated whole-body effects of exercise may be critical for resisting diabetes and obesity.

Obesity and type II diabetes are a major worldwide problem jeopardizing the health and the economy in many countries, and needing urgent intervention^1^. Type II diabetes while in itself dilapidating, also causes cardiovascular complications including retinopathy, nephropathy, cardiac myopathy and peripheral vascular disease (characterized by skeletal muscle angiopathy) exacerbating morbidity and mortality^2^,^3^,^4^. An effective intervention for the management of obesity, diabetes and associated cardiovascular complications is a lifestyle change containing dietary control and regular physical exercise^5^,^6^. Particularly, endurance-type exercise decreases obesity, protects against insulin resistance and type II diabetes, and mitigates cardiovascular pathology associated with diabetes^7^. Unfortunately, overt obesity and poor cardiovascular health and even motivational paucity for lifestyle change may hamper implementation of diet and exercise. In this regard, ability to pharmacologically mimic exercise and its benefits is recently gaining popularity^8^,^9^,^10^. However, lack of complete understanding as well as effectiveness of exercise mimetic molecular pathways especially in the context of obesity and diabetes has hindered the implementation of exercise mimesis through pharmacology^11^.

Skeletal muscle is a large organ system critical for energy homeostasis, and is a common site of impaired metabolism and insulin signaling in type II diabetes^12^,^13^. Endurance exercise has a major adaptive impact on the skeletal muscle that mainly includes a fiber type switch to an oxidative type muscle, increased mitochondrial biogenesis and metabolism, as well as increased angiogenesis and muscle perfusion, which overall are proposed to not only enhance fat burning, but also improve insulin delivery and action in the skeletal muscle^14^. Within the skeletal muscle, oxidative myofibers seem to be more efficient in glucose uptake and sensitive to insulin stimulation^15^,^16^. Additionally, exercise-trained muscle due to increased oxidative capacity does not accumulate toxic lipids such as diacylglycerols (DAG) and ceramides, which are known to interfere with insulin signaling^17^,^18^,^19^. While exercise has global physiological effects, and simultaneously recruits multiple metabolic and signaling pathways in muscle, it is not clear which particular molecular pathways can efficiently mitigate obesity and diabetes. In general, whether exercise-independent and pharmaceutically directed fiber type switch and muscle vascularization is necessary and sufficient to boost metabolic efficiency in obesity and mitigate diabetes is unclear.

Estrogen-related receptors (ERR) belong to nuclear receptor super-family and play a major role in mitochondrial biogenesis and metabolic regulation. Skeletal muscles highly express both ERRα and ERRγ, with these receptors serving an important role in muscle function. Lack of ERRα represses the expression of various aerobic metabolic genes in the skeletal muscle leading to decreased muscle fitness and exercise intolerance^20^,^21^. We have extensively studied the role of ERRγ in the skeletal muscle. ERRγ is preferentially expressed in oxidative and highly vascularized muscles^22^, and is induced by endurance training^23^. We found that muscle-specific over-expression of ERRγ induced glycolytic-to-oxidative fiber type switch, mitochondrial biogenesis and angiogenesis in the skeletal muscle^22^,^23^. ERRγ-mediated muscle remodeling in our studies led to increased aerobic metabolism and exercise tolerance in the transgenic mice. Recent loss-of-function studies have further supported the role of muscle ERRγ in governing aerobic capacity and exercise tolerance^24^.

Because muscle-specific modulation of ERRγ signaling leads to exercise-like effects in lean mice; in this study, we have investigated whether ERRγ-mediated oxidative fiber type switch and vascularization in the skeletal muscle can prevent obesity and insulin resistance in db/db mice, a genetic model of type II diabetes.

## Results

### Muscle-specific ERRγ over-expression induces oxidative switch in db/db mice

**Figure 1:**
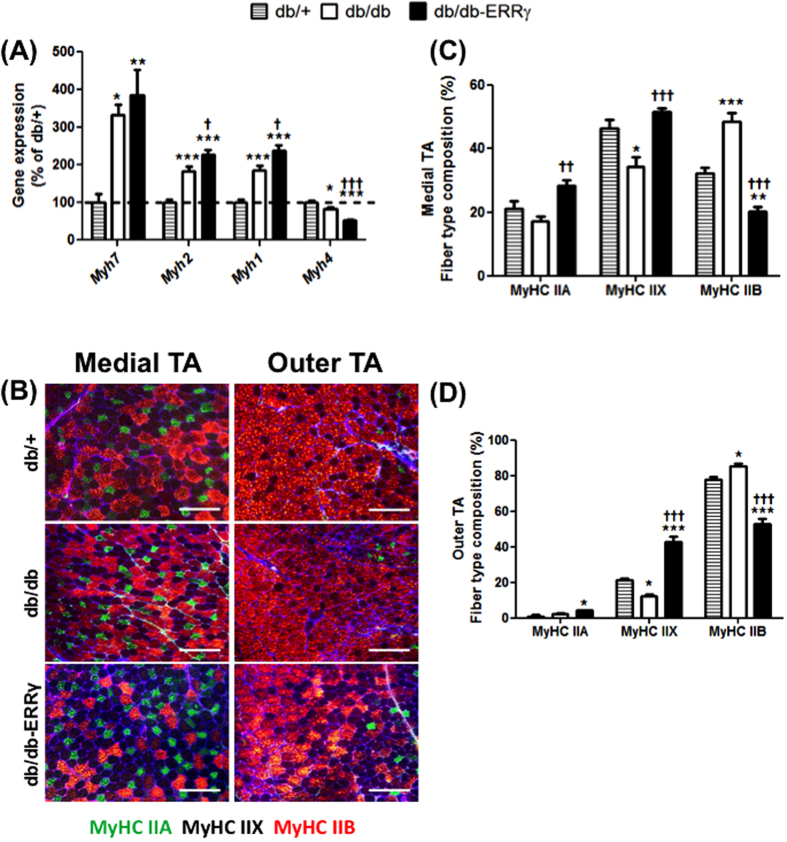
ERRγ changes myofiber type in db/db mice. Changes in myofiber type were measured in 6 month-old male non-fasted db/+ (hatched bars), db/db (open bars) and db/db-ERRγ (black bars) mice. **(A)** Myosin heavy chain (MyHC) gene expression in gastrocnemius muscles (N = 6–7). **(B–D)** Immunohistological myofiber type staining in Tibialis Anterior (TA) muscles for MyHC IIA (green), IIB (red), and IIX (unstained). **(B)** Representative images of the medial (left) and outer (right) TA cross-sections. **(C,D)** Percentage of positive fibers in the medial (**C**) and outer TA (**D**) (N = 5–6). *Indicates comparison to db/+ mice; ^†^indicates db/db compared with db/db-ERRγ mice. (*^/†^p < 0.05, **^/††^p < 0.01, ***^/†††^p < 0.001, One-way ANOVA with Tukey’s post-hoc test).

To examine the effect of muscle ERRγ in diabetes, we used db/db obese mice. We chose this genetic model mainly because (i) db/db mice develop severe obesity and diabetes, and (ii) because they exhibit skeletal muscle vascular regression^25^, which is not seen in diet-induced obesity/diabetes model, but is common in diabetic humans^26^,^27^,^28^. To over-express ERRγ specifically in the skeletal muscles of db/db mice, we crossed the muscle-specific ERRγ transgenic mice^22^ with the db/db mice. We confirmed the induction of both ERRγ gene and protein expression in the skeletal muscles of the db/db-ERRγ compared to the db/db control mice (Supplemental Fig. 1A,B). To determine the effect of ERRγ over-expression on muscle fiber type switch in db/db mice; we first measured the gene expression of MyHC (Myosin Heavy Chain) isoforms. The expression of Myh 7, 2 and 1 (representing oxidative type I, oxidative type IIA and oxidative/glycolytic IIX myofibers, respectively) were induced in db/db compared to db/+ mice (Fig. 1A). The expression of Myh 4 (representing glycolytic MyHC IIB myofibers) was decreased by 18% in db/db vs. db/+ mice (Fig. 1A). The expression of Myh 2, and 1 were further induced in db/db-ERRγ (by 20% and 22%, respectively) compared to db/db muscles; whereas, the expression of Myh 4 was further repressed (by 40%) (Fig. 1A). The expression of Myh 7 was not induced by ERRγ. Next, we used immunofluorescence to measure the proportions of different fiber types in both the outer glycolytic and medial oxidative Tibialis Anterior (TA) muscles (Fig. 1B–D). We found in both the outer and medial regions a decrease in MyHC IIX, and a reciprocal increase in MyHC IIB myofibers, in the db/db muscles compared to the db/+ muscles. In the db/db-ERRγ muscles, the proportions of MyHC IIA and/or IIX myofibers are increased compared to the db/db and the db/+ muscles; whereas, the percentage of MyHC IIB myofibers is decreased in db/db-ERRγ muscles (Fig. 1B–D). Collectively, these data indicates that diabetes causes an oxidative-to-glycolytic myofiber switch in the muscles, resulting in a compensatory increase in oxidative fiber type biomarker genes. Over-expression of ERRγ further induces oxidative myofiber biomarker genes and is able to retain a higher proportion of oxidative myofibers in diabetic mice.

### Muscle-specific ERRγ over-expression increases functional mitochondria content

**Figure 2:**
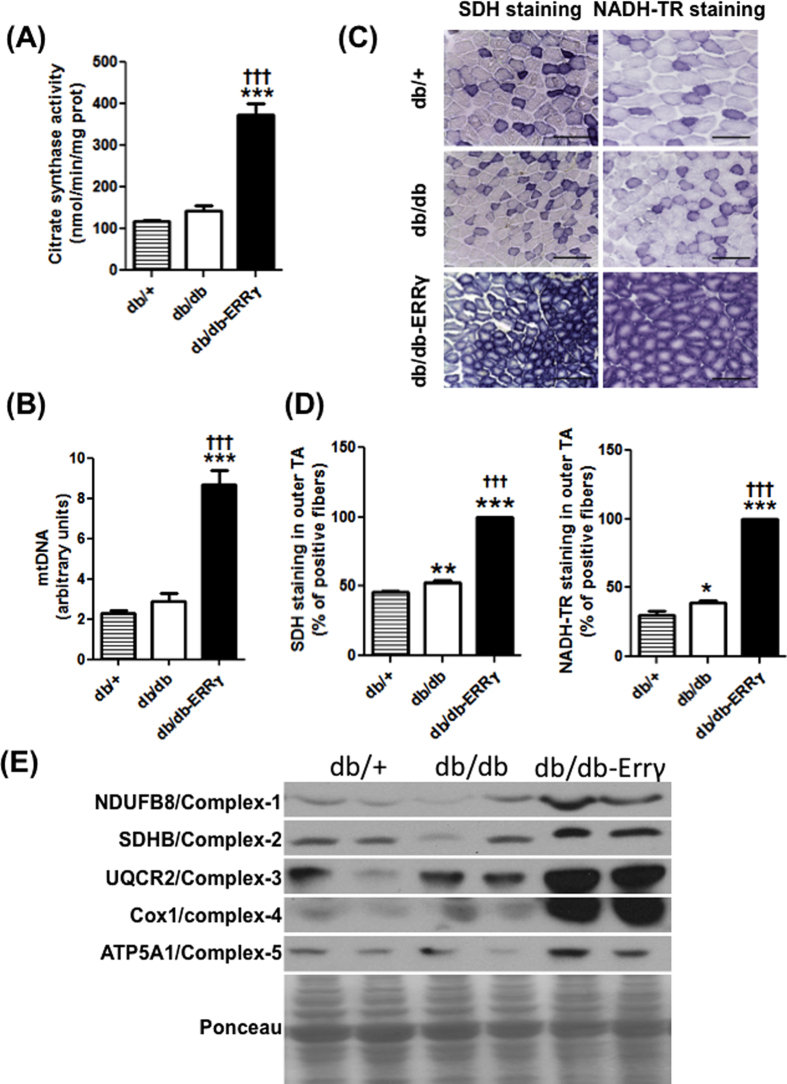
ERRγ induces mitochondrial gene expression and content in db/db mice. The following parameters were measured in 6 month-old male non-fasted db/+ (hatched bars), db/db (open bars) and db/db-ERRγ (black bars) mice. **(A)** Citrate synthase activity measured in biceps femoris muscles (N = 4). **(B)** Mitochondrial DNA content measured in biceps femoris muscles (N = 6–7). **(C,D)** Succinate dehydrogenase (SDH) and Nicotinamide Adenine Dinucleotide (NADH)-Tetrazolium Reductase (NADH-TR) staining in Tibialis Anterior (TA) muscles. **(C)** Representative images of the outer TA muscle. **(D)** Percentage of positive fibers in the outer TA stained positive for SDH (N = 5–6) and NADH-TR activity (N = 4–6). **(E)** Representative western blotting image of mitochondrial complex biomarker protein expression. *Indicates comparison to db/+ mice; ^†^indicates db/db compared with db/db-ERRγ mice. (*^/†^p < 0.05, **^/††^p < 0.01, ***^/†††^p < 0.001, One-way ANOVA with Tukey’s post-hoc test).

In order to fully characterize the extent of oxidative switch in db/db muscles as a result of ERRγ over-expression, we performed a thorough analysis of mitochondrial content and function. Citrate synthase activity, representative of mitochondrial content in the skeletal muscle was unchanged in db/db muscle compared to the db/+ muscles. Muscle ERRγ over-expression increased citrate synthase activity in db/db-ERRγ mice compared to the db/db mice (2.8 fold ↑) (Fig. 2A). Likewise, mitochondrial DNA (mtDNA) content was increased in the db/db-ERRγ mice compared to the db/+ (3.8 fold ↑) and db/db groups (3 fold ↑). However, a significant difference in mtDNA was not observed between db/+ and db/db (Fig. 2B). Using NADH-TR activity/staining (complex I) or SDH activity/staining (complex II), we found a drastic increase in the maximal mitochondrial activity in the db/db-ERRγ muscles compared to the two other groups (Fig. 2C,D and Supplemental Fig. 2B,C). Together with increased mtDNA, the data demonstrates an increase in mitochondrial content in the db/db muscles over-expressing ERRγ. Associated with the increase in mitochondrial content and activity, we also observed an increase in the expression of representative protein markers of mitochondrial respiratory complexes (NDUFB8, SDHB, UQCRC2, COX1, ATP5A1) in the db/db-ERRγ muscle (Fig. 2E and Supplemental Fig. 2A). These changes in mitochondrial content and respiratory complexes were associated with an increase in the expression of genes encoding mitochondrial proteins (*Cox5b, Atp5o, Nduf5a, Ucp3*) and of proteins involved in lipid metabolism (*Pnpla2, Abhd5, Lpl, Cd36, Cpt1b*) (Supplemental Fig. 3A,B).

**Figure 3:**
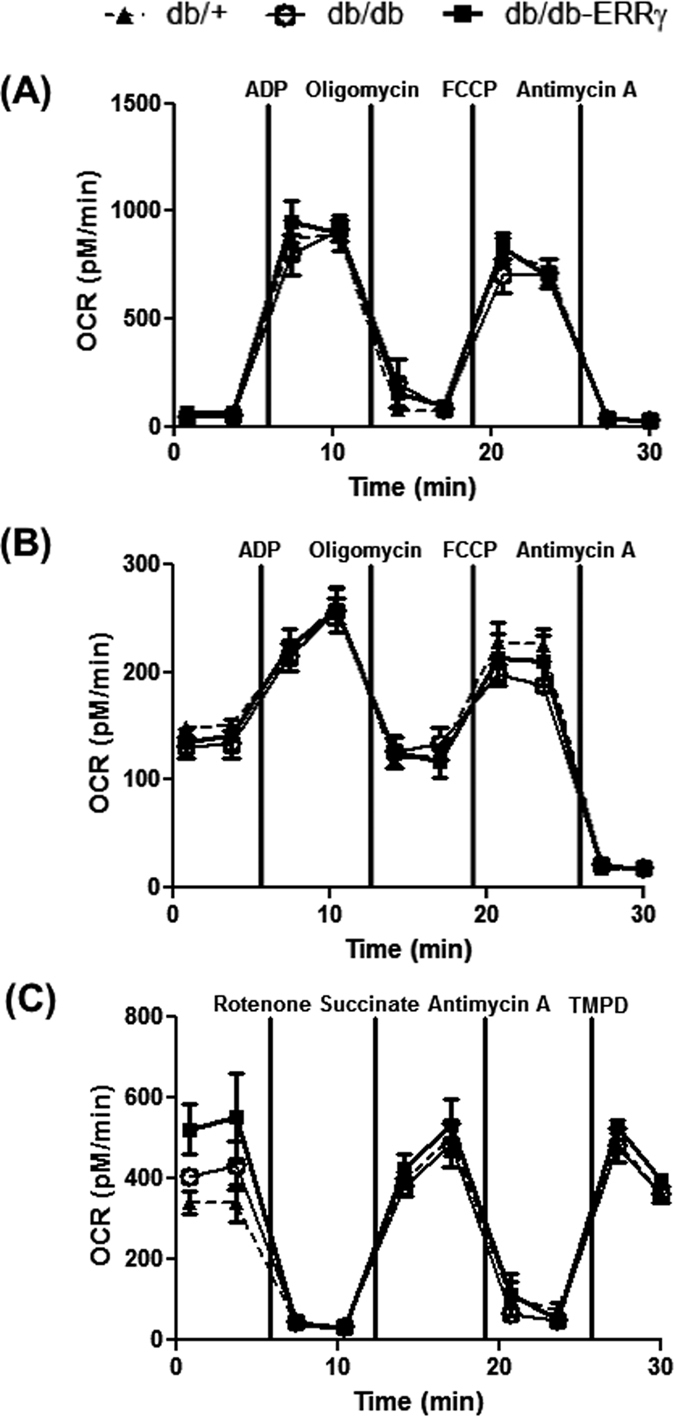
Oxygen consumption rate in isolated muscle mitochondria. The following parameters were measured in mitochondria isolated from muscles of 8 month-old male non-fasted db/+ (▴), db/db (⚪) and db/db-ERRγ (▪) mice. **(A,B)** Oxygen consumption rate (OCR) was measured in the presence of pyruvate/malate (**A**) or succinate/rotenone (**B**) in mitochondria treated sequentially with ADP, oligomycin, FCCP and antimycin A (N = 3). **(C)** OCR was measured in the presence of pyruvate/malate and FCCP in mitochondria treated sequentially with rotenone, succinate, antimycin A and TMPD/Ascorbate.

We further performed oxygen consumption studies on mitochondria isolated from db/+, db/db and db/db- ERRγ muscles to determine whether there were any inherent differences in mitochondrial function due to obesity or ERRγ overexpression. The functionality of the isolated mitochondria was determined by measuring oxygen consumption rate under different conditions using Seahorse. The baseline oxygen consumption rates of the mitochondrial preparations were comparable between the three groups, in presence of pyruvate/malate substrate (measuring complex I respiration) or succinate/rotenone (measuring complex II respiration) (Fig. 3A,B). Stimulation of oxygen consumption rates in state 3 (by ADP), state 4o (by oligomycin) or state 3u (by FCCP) were also comparable between groups either in presence of pyruvate/malate or succinate/rotenone (Fig. 3A,B). The uncoupled respiratory rates (in presence of FCCP) of complex I (using pyruvate/malate), complex II (using rotenone/succinate) and complex IV (using TMPD) were also comparable between the groups (Fig. 3C).

Collectively, these results demonstrate that there is no muscle mitochondrial dysfunction or decreased mitochondrial content in the obese db/db mice compared to their lean db/+ littermates. Furthermore, ERRγ over-expression in the muscles of db/db-ERRγ mice increases oxidative capacity and *functional* mitochondrial content compared to littermate db/db and db/+ mice.

### ERRγ over-expression in the muscle prevents muscle vascular rarefaction in db/db mice

**Figure 4:**
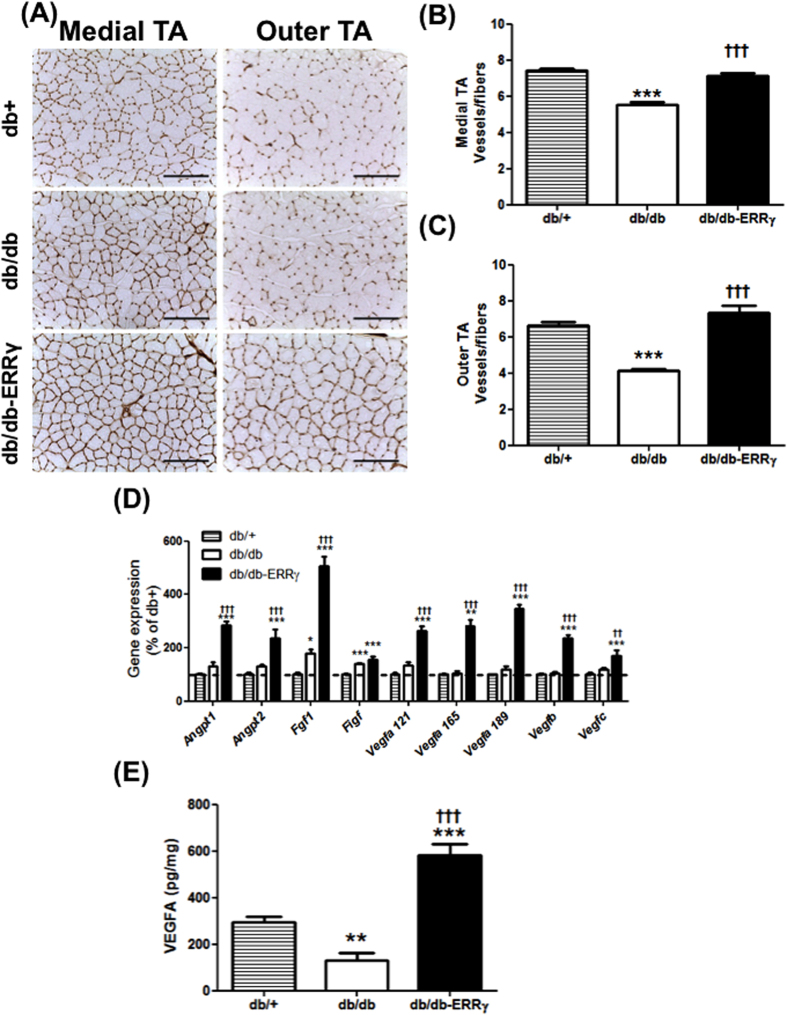
ERRγ induces angiogenic gene expression and capillary density in db/db mice. **(A)** Representative images of isolectin staining of capillaries in the medial and outer Tibialis Anterior (TA) of 6 month-old male db/+ (hatched bars), db/db (open bars) and db/db-ERR**γ** (black bars) mice. **(B,C)** Quantification of the number of vessels per fibers measured in the medial (**B**) and outer TA (**C**) (N = 4–6). **(D)** Relative expression of pro-angiogenic genes measured in the gastrocnemius muscles. (N = 4–8). **(E)** VEGFA protein content measure by ELISA in the gastrocnemius muscles. (N = 4–8). *Indicates comparison to db/+ mice; ^†^indicates db/db compared with db/db-ERRγ mice. (*^/†^p < 0.05, **^/††^p < 0.01, ***^/†††^p < 0.001, One-way ANOVA with Tukey’s post-test).

Diabetes causes vascular rarefaction, which decreases blood vessel density in the skeletal muscles, which may contribute to insulin resistance. Because ERRγ is a key regulator of many pro-angiogenic genes^22^,^29^,^30^, we measured whether ERRγ activates an angiogenic program and protects against vascular degeneration in db/db muscles. Vascular density was measured by capillary staining with isolectin, followed by quantifications (blood vessels-to-fiber ratio). As expected, the vessel-to-fiber ratio is decreased in the db/db muscles (in medial TA 25%↓ and in outer TA 38%↓ vs. the control db/+ muscles). The loss of muscle vasculature was prevented in the db/db-ERRγ muscles (in medial TA 29%↑ and outer TA 77%↑ vs. the db/db muscles) (Fig. 4A–C). The increase in muscle vasculature in the ERRγ overexpressing db/db mice was associated with an induction of a battery of pro-angiogenic genes, which was confirmed at the protein level for VEGFA – a master angiogenic regulator (Fig. 4D,E). Interestingly, many of the pro-angiogenic gene transcripts were normally expressed in diabetic muscles, suggesting deregulation in translation of these proteins, at least based on the observed decrease in VEGFA protein in the muscles of db/db compared to the db/+ mice.

### Muscle ERRγ activation does not prevent obesity or insulin resistance in db/db mice

**Figure 5:**
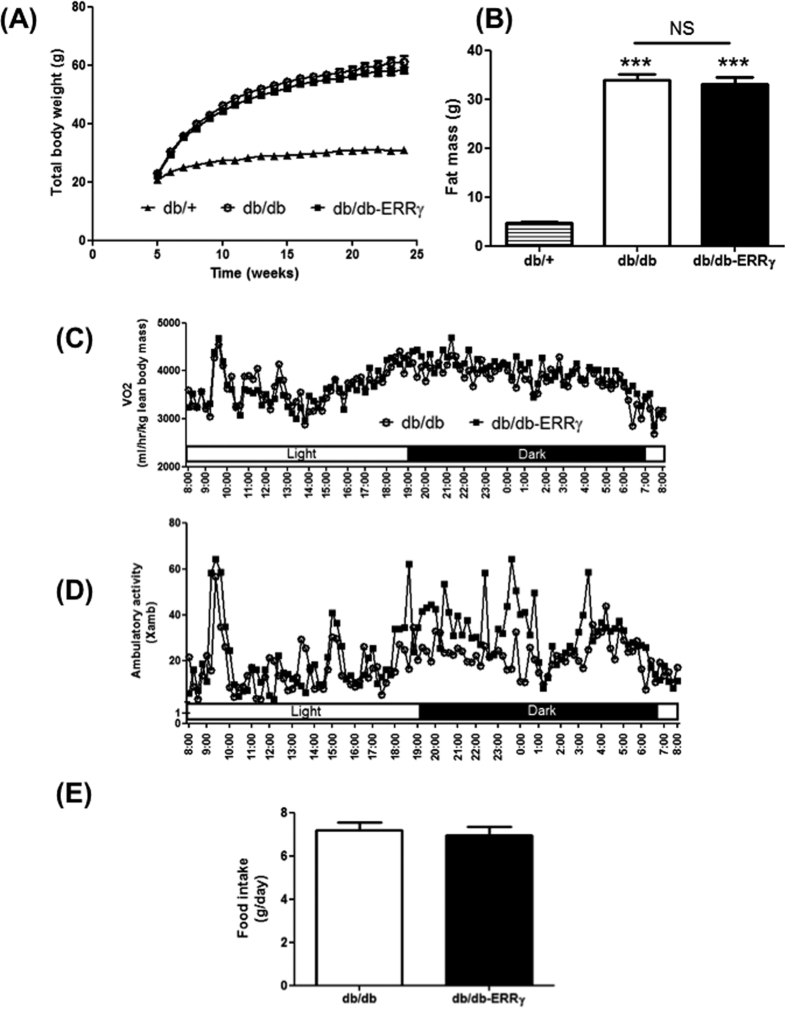
ERRγ does not increase energy expenditure in db/db mice. **(A)** Weekly measure of total body weight of male mice (N = 25–23). **(B)** Fat mass in 5 months-old male mice (N = 7–9). **(C–E)** Energy expenditure (**C**), ambulatory activity (**D**) and food intake (**E**) in 5 month-old male mice (N = 5–7). In (**A,C,D**): db/+ (▴), db/db (○) and db/db-ERRγ (▪). In (**B,E**): db/+ (hatched bar), db/db (open bar) and db/db-ERR**γ** (black bar). (***p < 0.001, One-way ANOVA with Tukey’s post-test).

We next investigated the effect of ERRγ-directed aerobic remodeling in the skeletal muscles on obesity and diabetes in the db/db mice. As expected db/db mice dramatically gained weight compared to the db/+ mice. Surprisingly and despite successful remodeling, ERRγ over-expression in the muscle failed to prevent the weight gain in db/db mice [db/+ = 10 g↑; db/db 38.55 g↑ (p < 0.001); db/db-ERRγ = 36.25 g↑ (p = NS)] (Fig. 5A). In association, we found a 7-fold (p < 0.001) increase in the fat mass in db/db compared to db/+ mice, but there was no change between the db/db and db/db-ERRγ mice [db/db = 32.4 g↑; db/db-ERRγ = 32.1 g↑ (p = NS)] (Fig. 5B). The lean body mass was slightly decreased in db/db compared to db/+ mice, but no change was observed between the db/db and db/db-ERRγ mice [db/+ = 25.1 g; db/db = 23.1 g (p < 0.05); db/db-ERRγ = 22.5 g (p = NS)]. Furthermore and unlike previous observation in the lean mice^22^, we did not detect any change in the energy expenditure (Fig. 5C), ambulatory activity (Fig. 5D), food intake (Fig. 5E), or respiratory exchange ratio (RER) (Supplemental Fig. 4) in db/db-ERRγ compared to the db/db mice.

**Figure 6:**
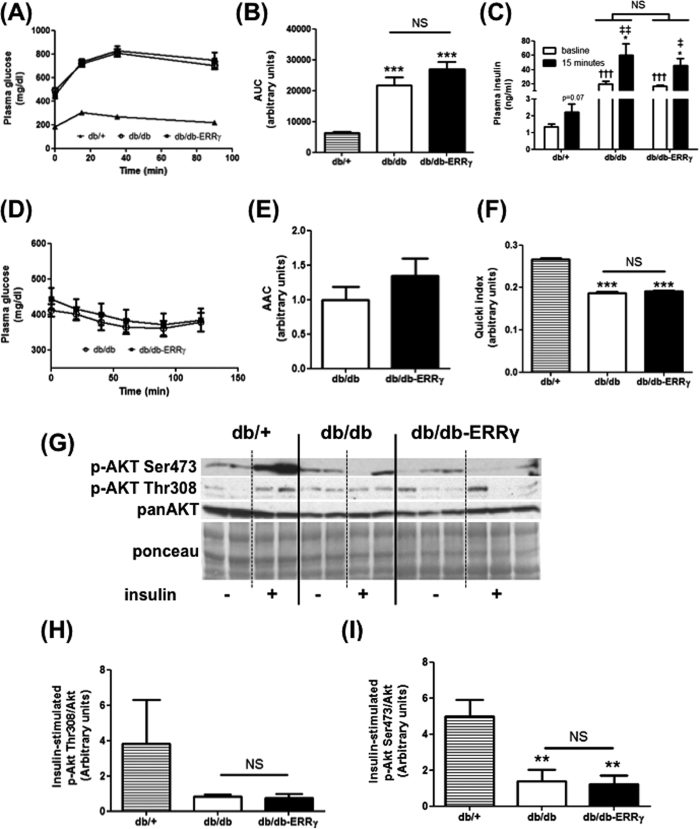
ERRγ does not mitigate insulin resistance in db/db mice. Following parameters are measured in db/+ (▴ or hatched bar), db/db (○ or open bar) and db/db-ERRγ (▪ or black bar) mice. **(A–C)** Glucose tolerance test performed on 4 month-old male mice (N = 6–8). Plasma glucose (**A**), Area Under the Curve (AUC) (B), and plasma insulin before and 15 min after glucose injection (**C**) were measured. **(D,E)** Insulin tolerance test performed on 4 month-old male mice (N = 11–12). Plasma glucose (**D**) and Area Above the Curve (**E**) were measured. **(F)** Quicki index values (N = 6–8). **(G–I)** Insulin signaling measured on *ex-vivo* insulin stimulated quadriceps muscles of 6 month-old male mice (N = 4–5). (**G**) Representative images. (**H**) Akt thr-308 phosphorylation. (**I**) Akt ser-473 phosphorylation. *Indicates comparison to db/+ mice; ^†^indicates db/db compared with db/db-ERRγ mice; ^‡^indicates baseline compared with t15 after glucose injection. (*p < 0.05, ****^/††/‡‡^p < 0.01, ***^/†††^p < 0.001, One-way ANOVA with Tukey’s post-test).

Next, we tested whether muscle-specific ERRγ activation protected against insulin resistance and glucose intolerance by performing insulin (ITT) and glucose (GTT) tolerance tests. We found a significantly impaired glucose tolerance in db/db vs. the db/+ mice (Fig. 6A). In the GTT, area under the curve (AUC) was dramatically increased in db/db mice vs. the db/+ mice (Fig. 6B). Furthermore, baseline and glucose-stimulated plasma insulin levels were elevated in db/db vs. the db/+ mice (Fig. 6C). However, these elevated parameters were not mitigated by over-expressing ERRγ in the skeletal muscles (Fig. 6A–C). Further, in ITT we did not observe any beneficial effect of ERRγ over-expression in muscle in comparing db/db vs. db/db-ERRγ mice (Fig. 6D,E). Using QUICKI index (a well-known marker of insulin sensitivity), we found a significant decrease of the QUICKI values in db/db mice compared to db/+ (31% decrease), showing that the mice are insulin resistant (Fig. 6F). No change was detected in QUICKI index between the db/db-ERRγ and db/db mice (Fig. 6F).

Analysis of the muscle insulin signaling pathway further confirmed that ERRγ over-expression did not protect against muscle insulin resistance. We detected a strong decrease in the insulin-stimulated phosphorylation of AKT at the reported ser-473 (3.6 fold less) and thr-308 (4.7 fold less, unpaired Student’s t test p = 0.06) activation sites in the muscles of db/db compared to db/+ mice (Fig. 6G). Activation of ERRγ in the muscle did not restore insulin-stimulated AKT phosphorylation at either of the sites (Fig. 6G, and quantified in Fig. 6H,I).

### Effects of muscle ERRγ activation on lipid clearance in db/db mice

**Figure 7:**
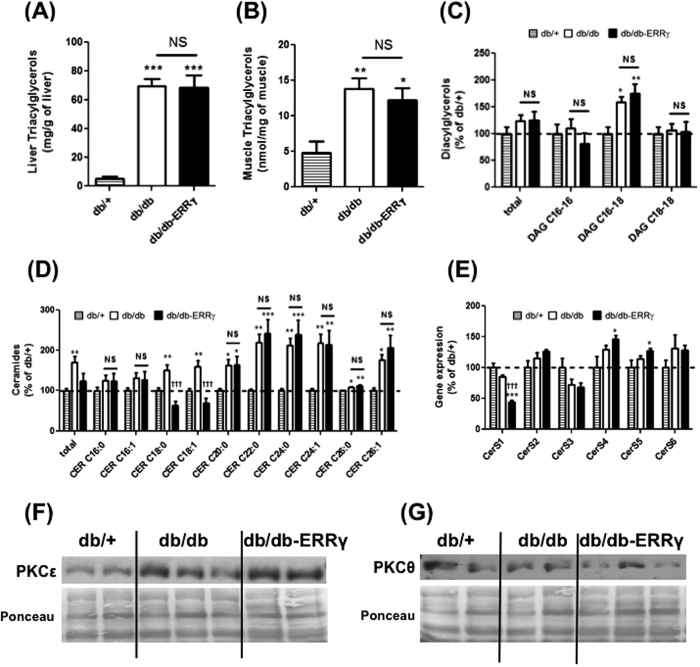
ERRγ effect on tissue lipid content and sarcolemmal PKC accumulation. Following measurements were made in 6 months-old fasted male db/+ (hatched bar), db/db (open bar) and db/db-ERRγ (black bar) mice. **(A,B)** Triacylglycerol (TAG) content measured in liver (N = 6–8) (**A**) and Tibialis Anterior muscle (TA) (**B**). **(C)** Diacylglycerols (DAG) species measured in the TA (N = 8). **(D)** Ceramide species measured in TA (n = 6–8). **(E)** Gene expression of ceramide synthase genes measured in the gastrocnemius (N = 6–4). (**F,G**) Representative images of sarcolemmal PKC accumulation for isoforms PKCε (**F**) and PKCθ (**G**) in quadriceps (N = 4–5). *Indicates comparison to db/+ mice; ^†^indicates db/db compared with db/db-ERRγ mice. (*p < 0.05, **p < 0.01, ***^/†††^p < 0.001, One-way ANOVA with Tukey’s post-hoc test).

Akin to exercise, and based on our previous studies, we expected ERRγ-mediated oxidative fiber type switch and angiogenesis in muscle to improve oxidative metabolism and fat burning and therefore enhance intramuscular lipid clearance, muscle insulin signaling and insulin sensitization. Because ERRγ failed to prevent either weight gain or insulin resistance, we asked whether ERRγ-mediated fiber type and angiogenic remodeling does actually decrease lipid accumulation in the skeletal muscles of db/db mice. To do so, we first measured the triacylglycerol (TAG) content in the liver and the muscles, two main organs involved in glucose homeostasis. While TAG content in liver (13 fold increase, p < 0.001) and muscle (2.8 fold increase, p < 0.01) were dramatically increased in the db/db mice compared to db/+ mice, muscle ERRγ activation failed to decrease TAG content in the liver and muscles of db/db mice (Fig. 7A,B), indicating the inability of muscle ERRγ activation to reduce lipid accumulation.

For further insight, we measured the muscle ceramides and DAG content, two well-known lipids suppressors of muscle insulin sensitivity in the skeletal muscle. We did not detect any change in total DAG in the muscles of db/db compared to db/+ mice. However, upon measuring individual species, DAG C16-18 was significantly increased (by 60%) in db/db muscles. Over-expression of ERRγ did not reduce the elevated DAG C16-18 content in the skeletal muscles of db/db mice (Fig. 7C).

In ceramide analysis, we found that several ceramide sub-species were increased in the db/db muscles compared to db/+ muscles, resulting in an increase in total ceramides. Interestingly, the total ceramide content showed a tendency to be decreased (unpaired Student’s t test p = 0.07) in db/db-ERRγ muscles compared to db/db muscles (Fig. 7D). However, this effect was only driven by a dramatic repression of CER C18 subspecies in db/db-ERRγ muscles even below levels seen in db/+ muscle (unpaired Student’s t test p < 0.05). The other sub-species remained all significantly elevated in db/db-ERRγ muscles (Fig. 7D). Ceramide synthesis is regulated by a group of ceramide synthases (CerS); where, each enzyme adds a distinct aryl chain to form a ceramide. We measured the expression of the six CerS in the 3 groups of mice. Interestingly, only the expression of the *CerS1*, which specifically controls CER C18 production was significantly repressed in the db/db-ERRγ muscles compared to the db/db muscles (2 fold, p < 0.001) (Fig. 7E). Therefore, the change in CER C18 levels was rather due to suppression of its synthesis by ERRγ than due to its breakdown.

Impaired insulin signaling in muscle is linked to increased sarcolemmal PKC (Protein Kinase C) expression, which results from myocellular lipid accumulation^18^. Therefore, we measured the sarcolemmal expression of the two major PKC isoforms (PKCε and PKCθ) known to impair insulin signaling in the skeletal muscle. Interestingly, while sarcolemmal PKCε was increased in db/db mice compared to db/+ (46%↑), sarcolemmal PKCθ was not affected (Fig. 7F,G). Furthermore, sarcolemmal localization of these PKC isoforms was not decreased by ERRγ over-expression in the db/db muscles (Fig. 7F,G).

Therefore, ERRγ does not prevent the accumulation of toxic lipid species in the diabetic skeletal muscles, which correlates with PKC sarcolemmal translocation and lack of insulin sensitization by ERRγ in the skeletal muscle.

## Discussion

In this study, we focused on the effect of ERRγ in the skeletal muscles of obese and diabetic mice because of its promising endurance exercise mimicking effects we observed in lean mice^22^,^29^. We report that muscle ERRγ over-expression indeed activates a gene program inducing glycolytic-to-oxidative switch, functional mitochondrial biogenesis and skeletal muscle vascularization, akin to exercise, in the muscles of db/db mice. Despite the exercise-like effects muscle ERRγ activation did not protect against obesity and diabetes. The lack of effect is likely due to insufficient substrate oxidation and lipid clearance, hampering weight loss and insulin sensitization in db/db mice. Therefore, ERRγ-driven fiber type switch and muscle vascularization may not be a strategy to mitigate obesity and diabetes.

Several pathways have now been described that are recruited during exercise to induce fiber type switch and vascular remodeling in the skeletal muscle. For example, the calcineurin signaling pathway was the first to be described to induce fiber type switch in skeletal muscle^31^. Members of the nuclear receptors and related co-factor family are also involved in skeletal muscle remodeling. Activation of nuclear receptor PPARδ and ERRγ induces glycolytic-to-oxidative switch, mitochondrial biogenesis and vascularization in the skeletal muscle^22^,^29^,^32^. Similarly, nuclear receptor co-activators PGC1α and PGC1β promote aerobic remodeling, whereas, other co-repressors such as RIP140 and NCOR1 are inhibitors of oxidative myofiber specification^33^,^34^,^35^,^36^,^37^. Whether all these pathways impact the progression of obesity and type II diabetes has not been fully elucidated. To our surprise, ERRγ-mediated oxidative fiber type switch, functional mitochondrial biogenesis and muscle vascularization did not prevent obesity and diabetes in the db/db mice. This is in sharp contrast to the effect of PPARδ in the skeletal muscle, which protected against weight gain and also mitigated insulin resistance^32^,^38^. Interestingly, activation of PGC1α (a nuclear receptor co-activator) in the skeletal muscle, which is proposed to function through ERR’s, also does not protect against weight gain and insulin resistance^10^,^39^,^40^. Moreover, inactivation of NCOR1 (a nuclear receptor co-repressor) in the skeletal muscle only mildly protected against insulin resistance, despite oxidative fiber type switch^36^. Collectively, in disagreement with the previous hypothesis, selectively mimicking fiber type and angiogenic switching effects of exercise may not be sufficient in the skeletal muscle for protection against metabolic disorders.

Clue as to why muscle ERRγ activation fails to induce anti-obesity and anti-diabetes effects arises from our metabolic studies. Clearly over-expression of ERRγ in muscle successfully activates the expected gene program involving stimulation of mitochondrial biogenesis, fiber type and angiogenic genes in obese mice. However, in db/db mice, ERRγ fails to impact energy expenditure, RER, weight gain, fat body mass or insulin resistance. Specifically, in the skeletal muscles of db/db mice activation of the ERRγ pathway does not prevent triglyceride accumulation. In addition, we measured two main lipid species, namely the DAG and the ceramides, which are known to impair muscle insulin sensitivity. Only one species of DAG was increased in the diabetic muscles, the DAG C16:18; however, ERRγ did not suppress its induction. Increase in DAG C16:18 was associated in our study with an increase in sarcolemmal PKCε in the diabetic muscles. Interestingly, we didn’t find in the db/db mice any sarcolemmal translocation of PKCθ, which is also known to promote insulin resistance^18^,^19^. Previous studies have linked an increase in sarcolemmal PKCθ with DAG C18:18 accumulation, which was not increased in these mice^41^. Nevertheless, DAG C16:18 and PKCε activation are both linked to muscle insulin resistance in humans^42^,^43^. The total ceramide content was dramatically increased in the diabetic muscle, and ERRγ over-expression showed a tendency to restore ceramide content back to baseline. However, the effect of transgene on total ceramide content was exclusively due to the suppression of CER C18, and all the other species of ceramide remained elevated in the db/db-ERRγ mice. The specific effect can be explained by the repression of *CerS1* gene expression in the db/db muscles by ERRγ, an enzyme necessary for CER C18 synthesis. Therefore, decrease in CER C18 is due to a decrease in its synthesis rather than due to its breakdown. Interestingly, insulin sensitivity was not affected in the db/db-ERRγ (compared with db/db mice) despite the low level of CER C18 indicating that CER C18 species may not be involved in the muscle insulin resistance. Furthermore, because other lipids (e.g. TAG, DAG and ceramides) collectively remain high in diabetic muscles with ERRγ over-expression, we suggest that the ERRγ-mediated skeletal muscle remodeling including increased mitochondrial biogenesis is not able to prevent the accumulation of lipids and lipotoxic species in the skeletal muscles of the diabetic db/db mice. Similar to our observations in this study, PGC1α and SIRT1 over-expression in muscle failed to increase energy expenditure and protection against obesity despite robustly increasing muscle mitochondrial content^10^,^39^,^40^,^44^.

The lack of effect of ERRγ over-expression or even the detrimental effects of obesity/diabetes on metabolic homeostasis does not seem to be linked to dysfunctional mitochondria in the skeletal muscles of db/db mice. Mitochondria isolated from the skeletal muscle exhibited comparable respiratory rates between db/+, db/db and db/db- ERRγ mice. Therefore, obesity or hyperglycemia does not seem to cause mitochondrial defect in the skeletal muscle. More importantly, mitochondrial biogenesis by ERRγ over-expression in db/db-ERRγ mice produces fully functional mitochondria.

AMPK is a critical driver of mitochondrial biogenesis, oxidative metabolism and insulin sensitization in the skeletal muscle. AMPK is also a downstream target of leptin signaling in the skeletal muscle^45^. Therefore, the lack of effect of muscle ERRγ activation on energy expenditure, weight gain and insulin signaling in db/db muscle could possibly be due to impaired leptin and/or AMPK signaling in db/db muscles. The p-AMPK (active form where thr-172 is phosphorylated) levels are indeed significantly decreased in db/db muscles compared to the db/+ muscles from our studies, likely due to the lack of leptin signaling (Supplemental Fig. 5). Despite the lack of leptin signaling, we found p-AMPK to be actually induced in db/db-ERRγ muscles compared to db/db muscles. Moreover, we found that muscle ERRγ over-expression is unable to improve glucose tolerance in mice on high-fat diet (data not shown). Therefore, the lack of ERRγ effect in db/db mice could not be due to impaired leptin and/or AMPK signaling. On the contrary, the increase in the AMPK activity could partially be responsible for the increase of the mitochondrial and vasculature content in the db/db-ERRγ mice.

We speculate that the likely reason for the absence of physiological effect on energy expenditure in case of muscle-specific- ERRγ could be the lack of sufficient mitochondrial activation or oxidative metabolism in obese db/db mice to counteract lipid burden, as previously demonstrated for PGC1α^40^.

Angiogenic factors and vascularization may facilitate insulin delivery and signaling in muscle^46^. For example, muscle-specific VEGFA deletion results in insulin resistance^47^, speculatively due to direct effect of VEGFA on insulin signaling via the AKT pathway or by regulating the vasculature. Nevertheless, induction of VEGFA expression or vascular supply by ERRγ does not seem to contribute to insulin sensitization or lipid clearance in the skeletal muscles of db/db mice. Likewise, PGC1α, albeit in independent studies, also increased skeletal muscle vasculature in diabetic mice^33^, but did not affect insulin sensitization^10^,^39^,^40^. Interestingly, several studies have demonstrated the importance of microvasculature and insulin-mediated capillary recruitment in glucose and insulin delivery to muscle^46^,^47^,^48^,^49^. Furthermore, insulin resistance in endothelial cells leading to endothelial dysfunction and inability of insulin as well as glucose to transit across endothelial cells into the muscle could also contributes to type II diabetes^46^. Our work and those of others suggests that while muscle regulators such as ERRγ and PGC1α enhance skeletal muscle vascularization in diabetic muscle, their beneficial effects might be limited if the new vasculature is dysfunctional.

Regular exercise is an effective intervention in the management of obesity and diabetes. Health benefits coincide with exercise-mediated remodeling of the skeletal muscle fiber type, metabolism and vasculature leading to improved metabolic efficiency. Conversely, exercise capacity might be decreased in obesity and diabetes due to cardiovascular and other complications, further exasperating the condition. In this context, identification of tissue-specific pathways that induce exercise-like effects to combat diabetes is of particular importance. Here we found that ERRγ, which is induced in the skeletal muscles by exercise^23^, and which promotes exercise-like fiber type and vascular remodeling in the muscle, fails to mitigate obesity and diabetes in db/db mice, likely due to lack of increase in energy expenditure and muscle lipid clearance. Given that newer factors regulating skeletal muscle fiber type and vasculature in healthy muscle are increasingly being discovered, our findings show that they may not sufficiently mimic exercise in mitigating obesity and diabetes. Consequently, efforts may need to be specifically focused on understanding how to muscle-specifically or globally improve lipid metabolism and clearance.

## Methods

### Mouse husbandry

Mice (db/+, db/db, and db/db-ERRγ strains) were bred and housed at the Brown Foundation Institute of Molecular Medicine’s vivarium. These mice were generated by crossing the db/db mice (Jackson Labs, Stock # 000697, B6.BKS(D)-*Lepr*^*db*^*/J*) with muscle-specific ERRγ transgenic mice, and littermates were always used in the experiments. Because the homozygous db/db mice are sterile, mating was always set between db/+ and (db/+)-ERRγ. Muscle-specific transgenic mice generated using human alpha-actin promoter, were previously described^22^. Because the heterogeneous db/+ mice have normal weight gain and glucose tolerance (The Jackson Labs), littermate db/+ mice from the breeding were used as controls to eliminate any strain-related variability, as has been routinely done^50^,^51^,^52^,^53^,^54^,^55^. The room temperature was kept between 20–22 °C under 12:12 hr light-dark cycle with free access to water and food and were fed ad libitum on chow diet (Pico-Lab rodent diet 20; 13.2% Fat). Cages were changed twice a week. Animals were maintained and treated in accordance with the U.S. National Institute of Health Guide for Care and Use of Laboratory Animals, and the Animal Welfare Committee at The University of Texas Medical School at Houston approved all the experimental procedures.

### Body mass and body composition

Body weight was measured weekly at the same time of the day. Body composition was measured using quantitative nuclear magnetic resonance imaging (EchoMRI 3-in-1 system).

### Tissue collection and preparation

Mice were euthanized by cervical dislocation after 6 hours of fasting and tissues were rapidly extracted. For RNA, muscles were freeze-clamped in liquid nitrogen. For insulin signaling experiments and muscle fractionation, quadriceps muscles were stimulated with insulin (1 μM) *ex-vivo*, as previously described^56^. For immunofluorescence Tibialis Anterior (TA) muscles were mounted in OCT and frozen in melting isopentane cooled down by liquid nitrogen.

### Gene expression

Total RNA was prepared using the Purelink Kit (Ambion). Total RNA was further reverse-transcribed to cDNA with SuperScript III Reverse Transcriptase (Invitrogen) and analyzed by quantitative real-time PCR on the Applied Biosystems SYBR Green PCR Master Mix with an ABI-7900 cycler (Applied Biosystems). List of primers used and sequences is provided in Supplemental Table 1.

### Protein analysis by WESTERN blotting and ELISA

Tissues were homogenized in Pierce IP Lysis buffer (Thermo Scientific) using a polytron instrument at 25,000 rpm. Further the lysates were pre-cleared at 16,000 g, 20 min at 4 °C; and the supernatants were store at −80 °C. The protein content was measured using the Pierce BCA protein assay kit (Thermo Scientific). Protein samples (40 μg) were run on 8 to 15% poly-acrylamide gels, transferred onto nitrocellulose membrane and incubated with the primary antibodies [anti-pAKT ser473, anti-pAKT Thr308, anti-AKT, and GAPDH (Cell Signaling Technology); anti-PKCε (Santa Cruz Biotech); anti-total OXPHOS (Abcam) and anti-ERRγ (kindly provided by Ron Evans, Salk Institute)]. VEGFA protein was measured in gastrocnemius lysate using ELISA kit (R&D Systems, Minneapolis, MN, USA).

### Cytosol membrane fractionation

Plasma membrane and cytosol fractions from 50–70 mg of quadriceps muscles were extracted, as previously described^56^.

### Immunohistology

Serial transverse cryosections (10 μm thickness) were obtained from the mid-section of the TA muscles. Frozen muscle sections were processed for capillary staining with biotinylated isolectin B4 (Vector Laboratories). Fiber typing was performed by staining of myosin heavy chains (MyHC) type IIA and IIB using the mouse monoclonal antibodies A4.74 and BF.F3, respectively (Developmental Studies Hybridoma Bank), as we previously described^29^,^30^, negative fibers were considered as type IIX. All primary antibodies were visualized using suitable Alexa Fluor secondary antibodies from Molecular Probes. Isolectin was visualized with DAB peroxidase substrate (Vector Laboratories). Negative control staining by omitting either the primary or the secondary antibody was included in all sets of experiments.

### Insulin and glucose tolerance test

Tests were performed as described. 3.5 U/kg of insulin (Sigma-Aldrich) was used for ITT^57^. For GTT, blood was collected in EDTA-coated tubes. Glucose and insulin was measured in plasma using colorimetry (Cayman Chemical) and ultrasensitive ELISA (ALPCO Diagnostics), respectively. AUC or AAC were calculated to account for differences in baseline fasting blood glucose concentrations, as previously described^58^. Based on the GTT data, the insulin sensitivity index QUICKI was measured using the formula 1/[log (fasting insulin μU/mL) + log (fasting glucose mg/dL)].

### Metabolic cages

Energy expenditure was measured by oxygen consumption by indirect calorimetry and normalized to the lean mass. Individually housed mice maintained on chow diet were placed at room temperature (20–22 °C) in chambers of a Comprehensive Lab Animal Monitoring System (CLAMS; Columbus Instruments International). Food and water were provided ad libitum. Data were collection during 48 hr after 3 days of acclimation. Food intake was measured every 24 hr during 3 days.

### Mitochondrial DNA

Biceps Femoris DNA was extracted using the Nucleospin Tissue kit (MACHEREY-NAGEL). The mitochondrial DNA content was determined, as previously described^59^.

### Citrate synthase activity

Citrate synthase activity was measured in biceps femoris, as previously described^60^.

### Muscle mitochondrial isolation and oxygen consumption experiments

Mitochondria from TA and gastrocnemius muscles from both hindlimb’s were extracted, using a previously described protocol with slight modifications^61^. Briefly, muscles were minced in a 10 mM EDTA/PBS solution and digested for 30 min in 5 ml of 10 mM EDTA and 0.05% Trypsin/PBS solution. The digested and subsequently washed muscles were homogenized in 4 ml of EB1 (75 mM sucrose, 0.2% fatty acid free bovine serum albumin, 20 mM HEPES, 10 mM EDTA, 215 mM D-mannitol, pH 7.4) at 750 rpm using a potter coupled to a drill press. Homogenates were centrifuged at 700 × g for 10 min at 4 °C. Next, the supernatant was centrifuged again at 10,000 × g for 10 min at 4 °C. The pellet was re-suspended in EB2 (75 mM sucrose, 20 mM HEPES, 215 mM D-mannitol and 3 mM EGTA, pH 7.4) and centrifuged at 8,000 × g for 10 min at 4 °C. The pellet corresponding to the isolated mitochondria was re-suspended in a minimal volume of EB2 and protein quantified using the Bradford assay (Bio-Rad).

Two tests were performed on the isolated mitochondria using the XFe96 Seahorse instrument, as previously described^62^. Briefly 25 μl/well of mitochondria in cold MAS (70 mM sucrose, 220 mM mannitol, 10 mM KH_2_PO_4_, 5 mM MgCl_2_, 2 mM HEPES, 1 mM EGTA and 0.2% (w/v) fatty acid-free BSA, pH 7.4) containing substrate was plated on ice in a XFe96 culture plate and centrifuged at 2,000 × g for 20 min at 4 °C. Then 155 μl/well of warm MAS containing the basal condition was added. For the coupling experiment the baseline condition was pyruvate/malate (10 mM/2 mM with 2 μg of mitochondria) or succinate/rotenone (10 mM/2 μM with 1 μg of mitochondria), followed by injections of ADP (6 mM), oligomycin (2.5 μg/ml), FCCP (12 μM) and Antimycin A (4 μM). For the electron flow experiment, the baseline condition was pyruvate/malate/FCCP (10 mM/2 mM/12 μM with 2 μg of mitochondria), followed by injections of rotenone (2 μM), succinate (10 mM), Antimycin A (4 μM) and Ascorbate/TMPD (10 mM/100 μM).

### Succinate Dehydrogenase (SDH) and Nicotinamide Adenine Dinucleotide (NADH)-Tetrazolium Reductase (TR) Staining

SDH and NADH-TR staining was performed on cryo-sections (10 micron) of TA muscles, as previously described^37^,^63^.

### Metabolite measurements

#### Liver triacylglycerols

Liver (100 mg) lipids were extracted, using a previously described method with slight modifications^64^. Triacylglycerols (TAG) was measured using a colorimetric assay (Sigma-Aldrich).

#### Muscle triacylglycerols, diacylglycerols and ceramides

DAG/TAG and ceramides were measured in 20 mg or 5 mg of TA muscle lysate, respectively, as previously described^65^,^66^.

### Statistics

All statistical analyses were performed using GraphPad Prism 5.0 for Windows (GraphPad Software Inc.). Normal distribution of the data was tested with Kolmogorov-Smirnov tests. One-way ANOVA with a Tukey’s multiple comparison post-hoc test or unpaired Student’s t-tests with or without Welch correction were performed to determine differences between groups. All values in figures are presented as mean ± SEM. Statistical significance was set at p < 0.05.

## Additional Information

**How to cite this article**: Badin, P.-M. *et al.* Exercise-like effects by Estrogen-related receptor-gamma in muscle do not prevent insulin resistance in db/db mice. *Sci. Rep.* **6**, 26442; doi: 10.1038/srep26442 (2016).

## Supplementary Material

Supplementary Information
