# Supplementary material for: Exercise-like effects by Estrogen-related receptor-gamma in muscle do not prevent insulin resistance in db/db mice
Source: Sci Rep. 2016 May 25;6:26442. doi: 10.1038/srep26442 (PMC4879541; doi:10.1038/srep26442)
Supplement: Supplementary Information [file srep26442-s1.pdf]

SUPPLEMENTAL INFORMATION

Exercise-like effects by Estrogen-related receptor-gamma in muscle do not prevent obesity and insulin resistance in db/db mice

Pierre-Marie Badin [1], Isabelle K. Vila [3], Danesh H. Sopariwala[1], Vikas Yadav [1, 6], Sabina Lorca[1], Katie Louche[5], Eun Ran Kim[1], Qingchun Tong[1], Min Sup Song [3, 4], Cedric Moro[5], Vihang A. Narkar [1, 2, 4, 7]

[1] Metabolic and Degenerative Diseases, Institute of Molecular Medicine and [2] Integrative Biology and Pharmacology, The University of Texas Medical School at Houston; [3] Molecular and Cellular Oncology, The University of Texas MD Anderson Cancer Center, Houston; [4] Graduate School of Biomedical Sciences at The University of Texas Health Science Center at Houston; [5] Institut National de la Santé et de la Recherche Médicale, Inserm UMR 1048, Institute of Metabolic and Cardiovascular Diseases, and Paul Sabatier University, Toulouse, France; [6] **Current Address:** Indian Institute of Technology, Indore, India.

LIST OF CONTENTS

Supplemental Figure 1. Overexpression of ERRγ in db/db muscle.

Supplemental Figure 2. Mitochondrial content in db/db ERRγ mice.

Supplemental Figure 3. Metabolic and mitochondrial gene expression.

Supplemental Figure 4. Respiratory Exchange Ratio (RER) measurement.

Supplemental Figure 5. p-AMPK expression in the muscle.

Supplemental Table 1. QPCR primer sequences.

FIGURE LEGENDS

Supplemental Figure 1. Overexpression of ERRγ in db/db muscle. (A) ERRγ gene expression measured in gastrocnemius muscle of 6 month-old male mice. (N=4-6). **(B)** Protein expression of ERRγ in gastrocnemius muscle of 6 month-old male mice (N=4-5). * Indicates comparison to db/+ mice; † indicates db/db compared with db/db-ERRγ mice. (*/†$p<0.05$, ***$p<0.001$, One-way ANOVA with Tukey's post-hoc test.)

Supplemental Figure 2. Mitochondrial content in db/db ERRγ mice. **(A)** Quantification of the western blots for mitochondrial complex proteins (N=4). **(B & C)** SDH and NADH-TR staining in medial TA muscles. **(B)** Representative images of the medial TA SDH and NADH-TR staining. **(C)** Percentage of myofibers in the medial TA stained positive for the SDH (N=5-6) and NADH-TR staining (N=4-6). *Indicates comparison to db/+ mice; † indicates db/db compared with db/db-ERRγ mice. (**/††$p<0.01$, ***/†††$p<0.001$, One-way ANOVA with Tukey's post-hoc test.)

Supplemental Figure 3. Metabolic and mitochondrial gene expression. (A-B) Metabolic (A) and the mitochondrial (B) gene expression measured by QPCR. The data were obtained on gastrocnemius of 6 month-old fasted male mice (N=6-7). db/+, db/db and db/db-ERRγ mice are represented by hatched, open and black bars, respectively. * Indicates comparison to db/+ mice; † indicates db/db

compared with db/db-ERRγ mice. (*/†p<0.05, **/††p<0.01, ***/†††p<0.001, One-way ANOVA with Tukey's post-hoc test).

Supplemental Figure 4. Respiratory Exchange Ratio (RER) measured in 5 month-old male db/db (open bars) and db/db-ERRγ (black bars) mice.

Supplemental Figure 5. P-AMPK expression in the muscle. Activating phosphorylation of AMPK on residue Thr-172 measured in gastrocnemius muscles of 6 month old male mice (N=4). dB/+, db/db and db/db-ERRγ mice are represented by hatched, open and black bars, respectively. *Indicates comparison to db/+ mice; † indicates db/db compared with db/db-ERRγ mice. (*/†p<0.05, One-way ANOVA with Tukey's post-hoc test).

Supplemental Table 1. QPCR primer list.

Supplemental Figure 1

(A)

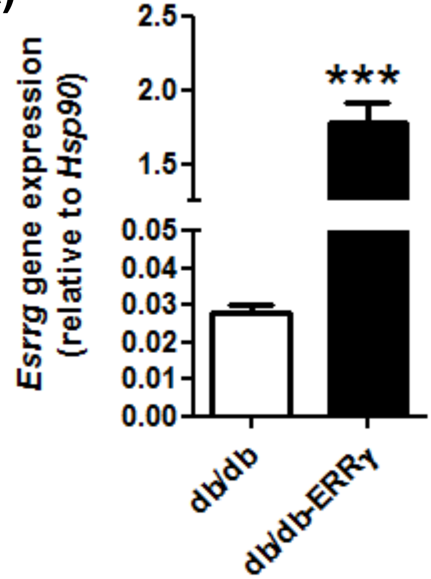

(B)

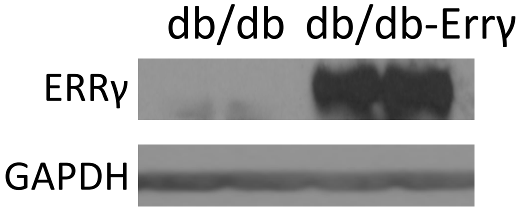

Supplemental Figure 2

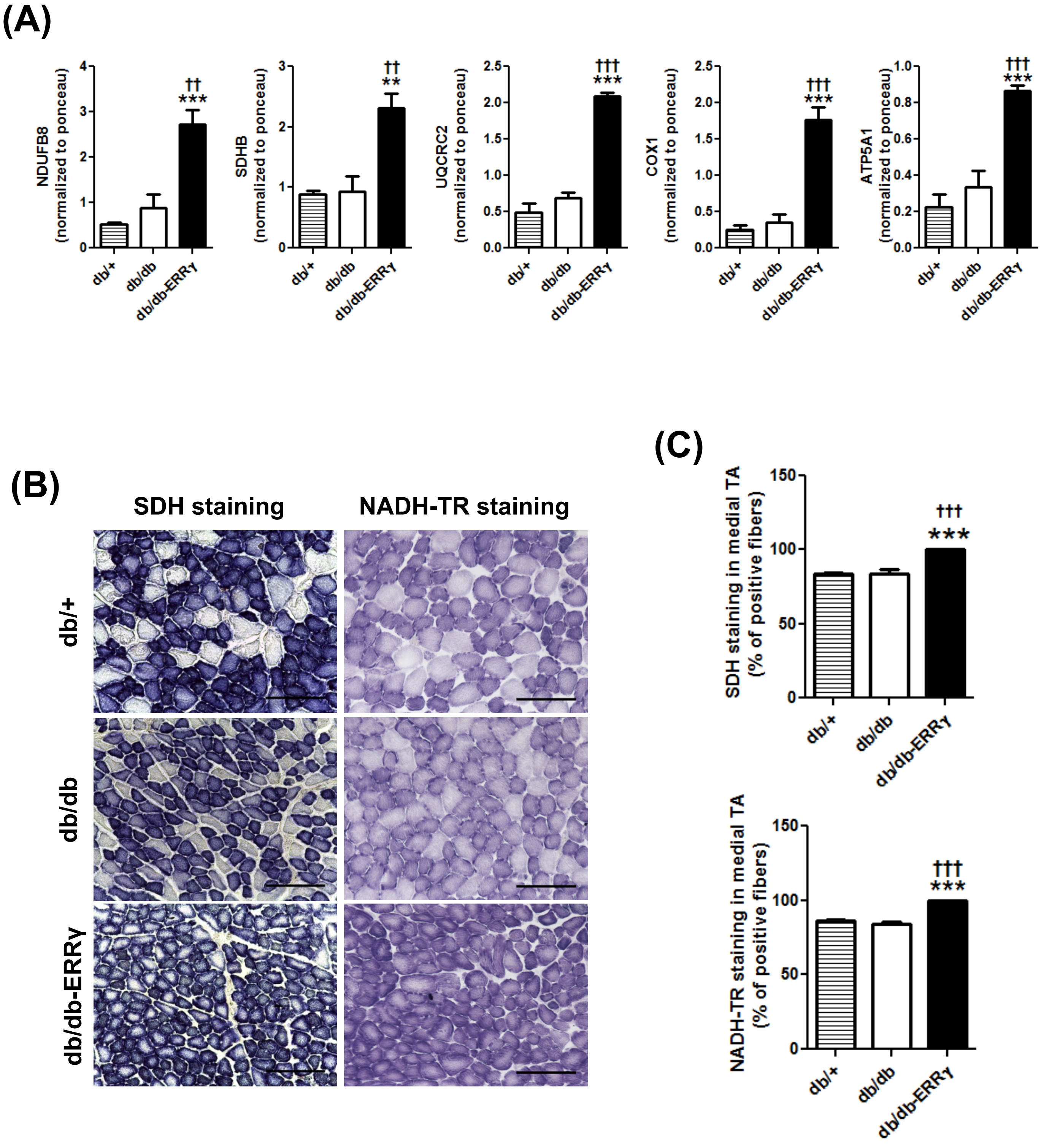

Supplemental Figure 3

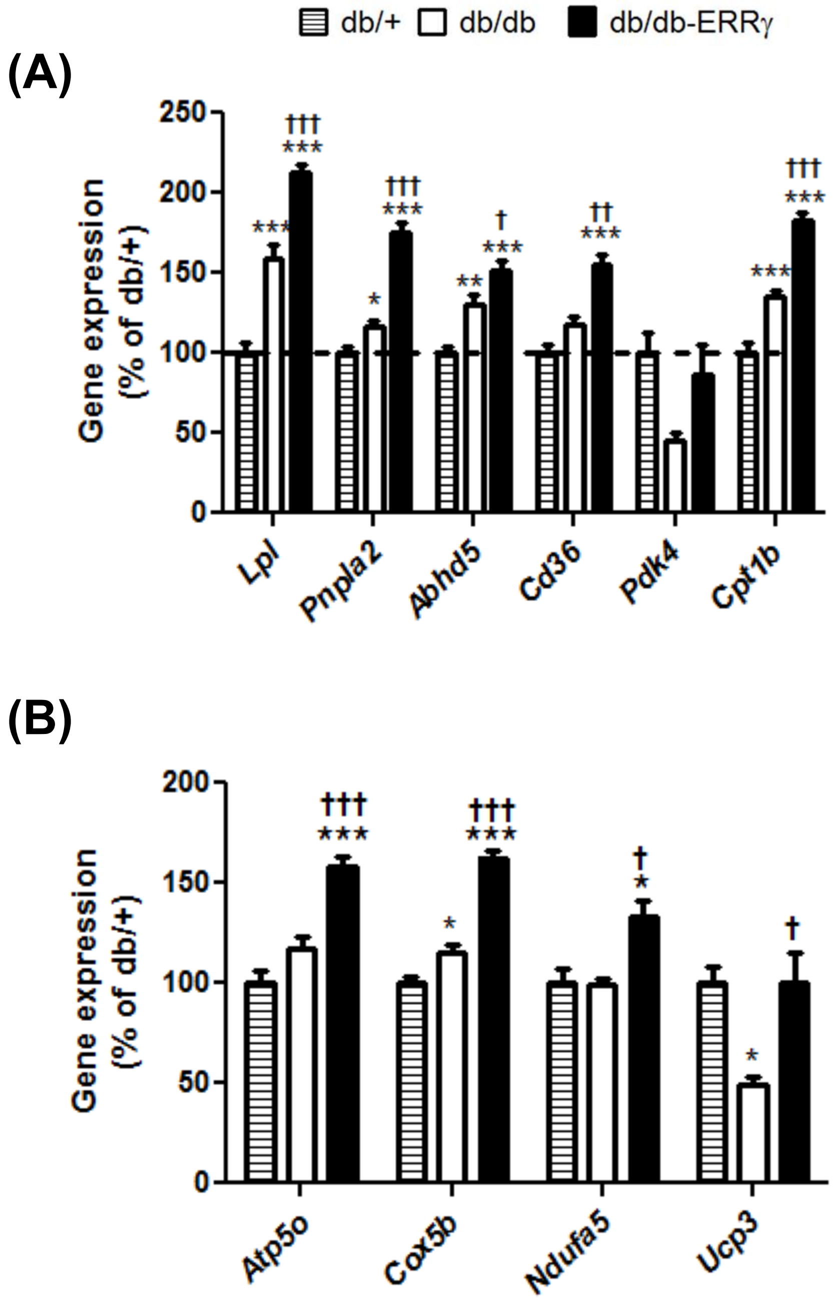

Supplemental Figure 4

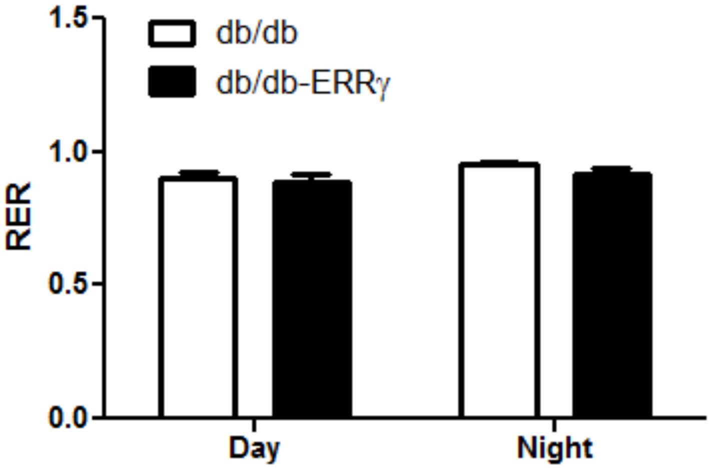

Supplemental Figure 5

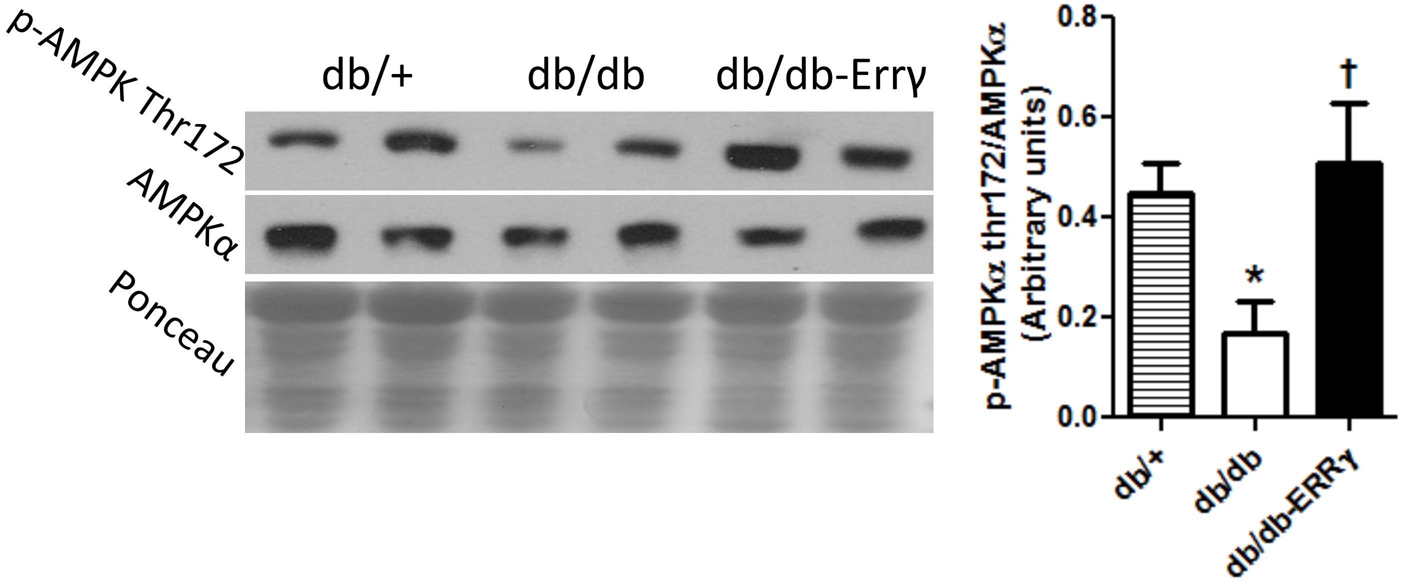

SUPPLEMENTAL TABLE 1

	Forward	Reverse
Abhd5	5’- TGACAGTGATGCGGAAGAAG-3’	5’-AGATCTGGTCGCTCAGGAAA-3’
Angpt1	5’-CATTCTTCGCTGCCATTCTG-3’	5’-GCACATTGCCCATGTTGAATC-3’
Angpt2	5’-GGTTGCTATCCGTAAAGAAGAGC-3’	5’-GGGGAAGGTCAGTGTGTAGATG-3’
Atp5o	5’-TCTCGACAGGTTCGGAGCTT-3’	5’-AGAGTACAGGGCGGTTGCATA-3’
CerS1	5’-GCCACCACACACATCTTTCGG-3’	5’-GGAGCAGGTAAGCGCAGTAG-3’
CerS2	5’-GAAGCCAGCTGGAGATTCAC-3’	5’-GACATCAGAGGCAATGCTGA-3’
CerS3	5’-CTCTGGGAGGTTTGGAATGA-3’	5’-CAGGTGGTGGATGACATGAG-3’
CerS4	5’-CCTGCTGGAGGTTTGTCTTC-3’	5’-GGCAAAGTGATCAGCAGTGA-3’
CerS5	5’-ATTTATTGCCAAGCCCTGTG-3’	5’-AACCAGCATTGGATTTTTCG-3’
CerS6	5’-CTGAAGAACACGGAGGAAGC-3’	5’-TATGGCACATGGTTTGGCTA-3’
Cd36	5’-CTGGGACCATTGGTGATGAAA-3’	5’-CACCACTCCAATCCCAAGTAAG-3’
Cox5b	5’-GCTGCATCTGTGAAGAGGACAAC-3’	5’-CAGCTTGTAATGGGTTCCACAGT-3’
Cpt1b	5’-GGGCACCCTCTGGGAGTTTGT-3’	5’-TGGCTCACCCACACAGTGT-3’
Esrrg	5’-ACTTGGCTGACCGAGAGTTG-3’	5’-GCCAGGGACAGTGTGGAGAA-3’
Fgf1	5’-GAAGCATGCGGAGAAGAACTG-3’	5’-CGAGGACCGCGCTTACAG-3’

Figf	5’-AAATCGCGCACTCTGAGGA-3’	5’-TGGCAAGACTTTTGAGCTTCAA-3’
Gapdh	5’-TGAAGCAGGCATCTGAGGG-3’	5’-CGAAGGTGGAAGAGTGGGAG-3’
Hsp90	5’-GTCCGCCGTGTGTTCATCAT-3’	5’-GCACTTCTTGACGATGTTCTTGC-3’
Lpl	5’- GCTGGGCCTAACTTTGAGTATG-3’	5’- CAAAATCAGCGTCATCAGGAGAA-3’
Myh1	5’-CTCCAGGCTGCTTTAGAGGAA -3’	5’-CCTGCTCCTAATCTCAGCATCC-3’
Myh2	5’-AATCTTACAAGAGACAAGCTGAGG-3’	5’-TGCGGAACTTGGATAGATTTG-3’
Myh4	5’- GAAGAGCCGAGAGGTTCACAC-3’	5’- CAGGACAGTGACAAAGAACGTC-3’
Myh7	5’-ACTGTCAACACTAAGAGGGTCA-3’	5’-TTGGATGATTTGATCTTCCAGGG-3’
Ndufa5	5’-AGCTGGATATGGTCAAGGCG -3’	5’- TGCTAACCAGTGGAAGTGGC -3’
Pdk4	5’-AAGCAAAACACAAACACGAGTA-3’	5’- CCCGGGTCATCCAACCA-3’
Pnpla2	5’- CAACGCCACTCACATCTACGG -3’	5’- GGACACCTCAATAATGTTGGCA -3’
Ucp3	5’-GAGATGGTGACCTACGACATCA-3’	5’-GCGTTCATGTATCGGGTCTTTA-3’
Vegfa 121	5’- TGCAGGCTGCTGTAACGATG-3’	5’- CCTTGGCTTGTCACATTTTTCT-3’
Vegfa 165	5’- TGCAGGCTGCTGTAACGATG-3’	5’-GAACAAGGCTCACAGTGATTTTCT-3’
Vegfa 189	5’- TGCAGGCTGCTGTAACGATG-3’	5’- CTCCAGGATTTAAACCGGGATT-3’

Vegfb	5’- TGCCATGGATAGACGTTTATGC-3’	5’- TGCTCAGAGGCACCACCAC-3’
Vegfc	5’- AAGACCGTGTGCGAATCGA-3’	5’- CACAGCGGCATACTTCTTCACT-3’
